# Revealing the Potential of *Fucus vesiculosus* Linnaeus for Cosmetic Purposes: Chemical Profile and Biological Activities of Commercial and Wild Samples

**DOI:** 10.3390/md22120548

**Published:** 2024-12-04

**Authors:** Marina Muñoz-Quintana, Carolina Padrón-Sanz, Marina Dolbeth, Francisco Arenas, Vitor Vasconcelos, Graciliana Lopes

**Affiliations:** 1 Faculty of Veterinary and Experimental Sciences, Catholic University of Valencia “San Vicente Mártir”, Guillem de Castro 94, 46001 Valencia, Spain; marinamquintana@gmail.com; 2 Translational Research Center San Alberto Magno (CITSAM), Catholic University of Valencia “San Vicente Mártir”, C/Quevedo, 2, 46001 Valencia, Spain; 3 Interdisciplinary Center of Marine and Environmental Research (CIIMAR/CIMAR), University of Porto, Terminal de Cruzeiros do Porto de Leixões, Av. General Norton de Matos s/n, 4450-208 Matosinhos, Portugal; mdolbeth@ciimar.up.pt (M.D.); farenas@ciimar.up.pt (F.A.); vmvascon@fc.up.pt (V.V.); 4 Faculty of Sciences, University of Porto, Rua do Campo Alegre, Edifício FC4, 4169-007 Porto, Portugal

**Keywords:** antioxidant, anti-inflammatory, fucoxanthin, tyrosinase, cosmetics

## Abstract

The natural products industry is gaining increasing interest, not only due to modern lifestyles becoming more aware of environmental and sustainability issues but also because of the loss of efficacy and undesirable side effects of synthetic ingredients. This pioneering study provides a comprehensive comparison between extracts obtained from wild and commercial samples of *Fucus vesiculosus* Linnaeus, highlighting their multifaceted benefits in cosmetic applications. The antiaging potential of acetone (70 and 90%) and ethanol 60% extracts from wild and commercial samples of *F. vesiculosus*, focusing on their application in cosmetics, was explored. The extracts were chemically characterized, their carotenoid profiles being established by HPLC, and the total phenolic content and phlorotannins by spectrophotometry. The extracts were evaluated for their antioxidant potential against the physiologic free radicals superoxide anion radical (O_2_^•−^) and nitric oxide (^•^NO), for their ability to inhibit the enzymes hyaluronidase and tyrosinase, and for their anti-inflammatory potential in the macrophage cell model RAW 264.7. The acetone 70% extract of wild *F. vesiculosus* was the richest in fucoxanthin, which accounted for more than 67% of the total pigments identified, followed by the acetone 90% extract of the same sample, where both fucoxanthin and pheophytin-*a* represented 40% of the total pigments. The same behavior was observed for phenolic compounds, with the ethanol 60% presenting the lowest values. A chemical correlation could be established between the chemical composition and the biological activities, with acetone extracts from the wild *F. vesiculosus*, richer in fucoxanthin and phlorotannins, standing out as natural ingredients with anti-aging potential. Acetone 90% can be highlighted as the most effective extraction solvent, their extracts presenting the highest radicals scavenging capacity, ability to inhibit tyrosinase to a greater extent than the commercial ingredient kojic acid, and potential to slow down the inflammatory process.

## 1. Introduction

Cosmetics have played an important role in human culture since ancient times. Initially derived from fruits, plants, and minerals, cosmetics were mostly used for religious and ornamental purposes [1].

Nowadays, the cosmetics business is one of the most significant and rapidly expanding sectors of the global economy, with a global revenue of USD 100.49 billion [2]. The cosmetics industry is anticipated to grow even more; in fact, according to recent projections, its European market will grow at a Compounded Average Growth Rate (CAGR) of 2.6% between 2022 and 2027 [3], and its global market will grow at a CAGR of roughly 5.5% between 2021 and 2028 [4]. Based on this, the market is demanding innovative, greener, and more sustainable solutions, in addition to increasing the use of natural products, due to the growing interest of consumers, industries, organizations, and the scientific community in reducing environmental impacts [5,6].

One of the most interesting and explored cosmetic properties related to skin aging is antioxidant activity, which is related to oxidative stress, a complex process involving internal and external factors (environmental variables such as pollution and UV radiation) where reactive oxygen species (ROS) and reactive nitrogen species (RNS) play an important role [7,8,9,10]. Consequently, during the previous several years, oxidative stress research has advanced quickly, and both the scientific community and the population have become more conscious of the importance of antioxidants [11,12].

Synthetic ingredients have been employed for many years; however, they have drawbacks, such as limited absorption rates and negative side effects [13,14]. Thus, as a consequence of modern lifestyle demands and the loss of efficacy of these products, natural bioactive substances are preferred [14,15]. In this context, seaweeds appear as rich sources of natural bioactive compounds with a variety of biological actions, making them suitable as active ingredients in cosmetic formulations [14,16,17,18]. Algal extracts contain a wide range of bioactive molecules from different classes: proteins, peptides, carbohydrates, minerals, iodine, lipids (PUFAs), phenols (polyphenols, tocopherols), alkaloids, terpenes, and pigments (chlorophylls, carotenoids, and phycobilins) that make them suitable for many purposes [12,19]. Among these components, one of the most promising applications is the development of cosmeceuticals and skin-care products [14].

In fact, macroalgae cultivation has expanded to answer the growing demand for the use of seaweed extracts for cosmetic formulations for sensitive skin, among others [20]. Seaweed bioactive extracts are employed as active ingredients in skin-whitening, anti-aging, colorants, thickening/gelling agents, or moisturizers [12,21,22]. Additionally, in some regions, seaweeds are used as an alternative therapy for skin-related disorders such as psoriasis and dermatitis [23,24], making them excellent natural raw materials for cosmetics with multiple benefits for health [15,16,25,26]. Among them, brown seaweeds occupy a prominent place for health and cosmetics due to their high antioxidant and anti-inflammatory activities, among others [27]. Particularly, some species of brown algae, such as *Fucus vesiculosus* Linnaeus, gained interest in recent years due to their abundance and significant commercial value as sources of natural ingredients with cosmetic and cosmeceutical applications [28,29,30]. Among the most interesting bioactive compounds of this species are pigments (chlorophylls and carotenoids) and polyphenols, more precisely phlorotannins [14]. Furthermore, the inhibitory effects of *F. vesiculosus* bioactive compounds on enzymes related to skin aging and the non-enzymatic glycation process were studied, proving effective against these processes and therefore demonstrating their potential to be employed as antioxidants and skin-lightening agents [31].

However, despite studies showing the high potential of the *Fucus* species in the cosmetics sector, it is known that the conditions in which they are collected, such as the season, can significantly influence their composition and, consequently, bioactivity [32,33]. To our knowledge, few studies have been conducted comparing the chemical composition and biological activities of wild and commercial seaweeds. Therefore, the objective of this work is to explore and compare the potential of different extracts obtained from wild and commercial *F. vesiculosus* for their cosmetic applications by evaluating their anti-inflammatory potential and their ability to inhibit key enzymes involved in the inflammatory process and scavenge physiologic free radicals, establishing a relationship between their chemical composition and bioactivity.

## 2. Results

### 2.1. Phytochemical Characterization

#### 2.1.1. Pigments Profile

**Figure 1:**
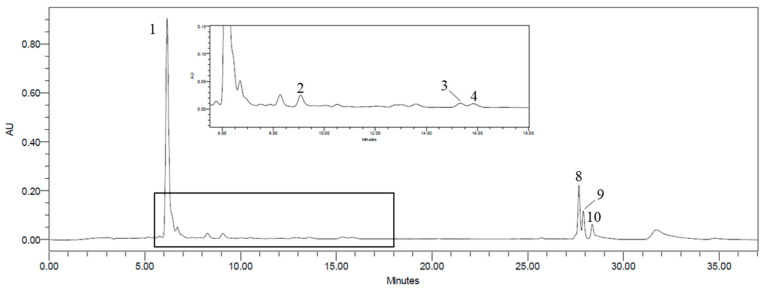
Carotenoid and chlorophylls profile of acetone 90% extract of the wild *Fucus vesiculosus*. HPLC-PDA recorded at 450 nm. Fucoxanthin (**1**), violaxanthin isomer (**2**), chlorophyll-*a* (**3**), zeaxanthin (**4**), β-carotene (**8**), pheophytin-*a* (**9**), and β-carotene derivative (**10**).

The carotenoid and chlorophylls profile of the different extracts, obtained from wild and commercial *F. vesiculosus* by HPLC-PDA, revealed the presence of four carotenoids, one chlorophyll derivative, chlorophyll *a*, and pheophytin *a* in the wild samples, and three carotenoids, eight chlorophyll derivatives, and pheophytin-*a* in the commercial ones (Table 1). The identified compounds for wild extracts included fucoxanthin (**1**), a violaxanthin isomer (**2**), β-carotene (**8**), β-carotene derivative (**10**), and zeaxanthin (**4**) among the carotenoids; and chlorophyll-*a* (**3**), chlorophyll-*a* derivative (**7**), and pheophytin-*a* (**9**) among the chlorophylls. For commercial extracts, the carotenoids identified were fucoxanthin (**1**), zeaxanthin (**4**), β-carotene (**8**), and β-carotene derivative (**10**); and the chlorophylls were 9 chlorophyll derivatives (**5**, **6**, **7**, **10**, **11**, **12**, **13**, **14**, **15**) and pheophytin-*a* (**9**). The chromatographic profile of wild *F. vesiculosus* acetone 90% extract is illustrated in Figure 1.

Fucoxanthin (1) was one of the main pigments quantified in all the extracts, with significant differences being observed for the extracts obtained from the wild *F. vesiculosus*: acetone 70% had the highest concentration (7067.12 ± 302.7 µg/g DE) followed by acetone 90% (3857.02 ± 149.06 µg/g DE) and ethanol 60% extract (2046.61 ± 107.20 µg/g DE). Regarding commercial samples, no significant differences were observed for the amount of this compound with the different extraction solvents, its concentration being much lower compared with the extracts obtained from the wild sample.

For the extracts obtained from the wild sample, total carotenoid concentrations ranged between 2046.61 and 7082.05 µg/g of dry extract (DE), with acetone 70% being the richest and ethanol 60% the poorest extract. Regarding chlorophylls, their concentration ranged between 3412.77 and 5095.36 µg/g DE, with ethanol 60% extracts presenting the highest amount and acetone 70% the lowest (*p* < 0.05). All the extracts of the wild *F. vesiculosus* contained fucoxanthin (**1**), β-carotene (**8**), and pheophytin-*a* (**9**), with chlorophyll-*a* (**3**) only being present in acetonic 70% and 90% extracts. Acetone 70% extract was the richest in fucoxanthin and acetone 90% in pheophytin-*a* (Table 1). The extracts obtained from the commercial *F. vesiculosus* were significantly the poorest in all the pigments analyzed. Total carotenoids ranged from 3.24 to 159.13 µg/g DE. Chlorophylls were dominant over carotenoids, ranging between 273.70 and 1987.58 µg/g DE in acetone 70% and acetone 90% extracts, respectively (*p* < 0.05). All the extracts of the commercial sample contained fucoxanthin (**1**), this being the only pigment found in the ethanolic extract. As expected, the results showed that extracts obtained from the wild sample are richer in both carotenoids and chlorophylls, which can be explained, at least in part, by the sample processing methods, e.g., lyophilization for the wild samples versus less conservative methods with higher exposure to oxygen during the drying process of the commercial *F. vesiculosus*. Additionally, the time that elapsed between the collection, drying, and analysis of the commercial samples could also lead to the degradation of some metabolites and consequently affect their chemical profile.

Although the amounts of carotenoids and chlorophylls were lower in the commercial sample, the high percentage of chlorophyll derivatives (**5**, **6**, **10**, **11**, **12**, **13**, **14**, **15**) in acetone 90% extracts must be highlighted. Among all the samples analyzed, wild ones were richer in both chlorophylls and carotenoids. Comparing the different solvents, acetone 90% must be highlighted for its high amount of β-carotene (**8**), fucoxanthin (**1**), and pheophytin-*a*; acetone 70% extracts stand out for their significant amount of chlorophyll-*a* (**3**) and fucoxanthin (**1**). Ethanol 60% extracts were the poorest, with only the major compounds (fucoxanthin, β-carotene, and pheophytin-*a*) being quantified.

#### 2.1.2. Total Phenolic Content (TPC) and Total Phlorotannin Content (TPhC)

The results of TPC and TPhC are displayed in Table 2.

Extracts from the wild *F. vesiculosus* presented higher TPC values compared to commercial extracts. Among them, the highest TPC was found in acetone 90%, followed by acetone 70% and ethanol 60% extracts (1903.83, 1042.75, and 792.28 µg/mg, respectively) (*p* < 0.05). Extracts of the commercial samples had the same pattern, with acetone 90% extract presenting the highest TPC and ethanol 60% the lowest (723.48 and 361.20 µg/mg, respectively) (*p* < 0.05). Regarding TPhC, the highest values were found in the acetone 90% extract of the wild sample (15.91 µg/mg), with the extracts of the commercial ones presenting lower values (5.42 µg/mg) (*p* < 0.05). Phlorotannins were below the limit of quantification in the ethanol 60% extracts of the commercial sample.

### 2.2. Antioxidant Capacity

**Figure 2:**
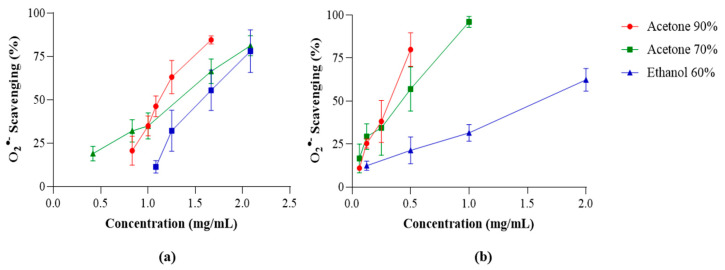
Superoxide anion radical (O_2_^•−^) scavenging activity of extracts obtained from commercial (**a**) and wild (**b**) *Fucus vesiculosus* samples. Values are expressed as the mean ± SD of at least three independent experiments, performed in duplicate.

The dose-dependent O_2_^•−^ scavenging activity of the extracts obtained from commercial and wild *F. vesiculosus* samples are displayed in Figure 2, while the IC_50_ values are presented in Table 3. Generally, the extracts obtained from commercial samples revealed weak O_2_^•−^ scavenging ability, while those obtained from wild ones were significantly more effective.

In wild samples, acetone 90% and acetone 70% extracts were the most effective, showing the lowest IC_50_ values (0.30 and 0.39 mg/mL, respectively), while in commercial samples the most effective extracts were acetone 90% and ethanol 60% (1.15 and 1.23 mg/mL, respectively). Comparing wild and commercial samples, there are significant differences in the three extraction solvents mentioned: acetone 90%, acetone 70%, and ethanol 60% (*p* < 0.05).

A negative correlation can be established between the IC_50_ values obtained for O_2_^•−^ scavenging, TPC (*p* < 0.01), and TPhC (*p* < 0.05), suggesting that these compounds contribute to the free radical scavenging (Table 2, Table 3 and Table 4). Moreover, there is also a negative correlation between O_2_^•−^ scavenging and carotenoids, such as fucoxanthin (*p* < 0.01).

**Figure 3:**
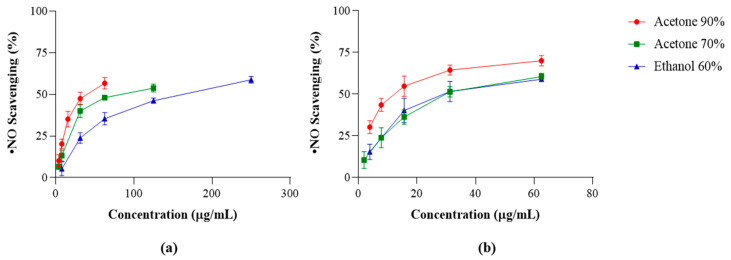
Nitric oxide radical (^•^NO) scavenging activity of extracts obtained from commercial (**a**) and wild (**b**) *Fucus vesiculosus* samples. Values are expressed as the mean ± SD of at least three independent experiments, performed in duplicate.

The ^•^NO scavenging activity of the extracts obtained from commercial and wild *F. vesiculosus* samples is displayed in Figure 3, while the respective IC_50_ values are presented in Table 3.

All the extracts analyzed presented a dose-dependent behavior. However, the extracts of the commercial *F. vesiculosus* revealed weaker ^•^NO scavenging activity compared to the wild ones, which were significantly more effective in reaching lower IC_50_ values. The acetonic extracts were the most effective, with acetone 90% presenting the lowest IC_50_ for both commercial and wild samples (38.22 and 12.09 µg DE/mL, respectively; *p* = 0.003), closely followed by acetone 70% extracts (58.63 and 29.02 µg DE/mL, respectively; *p* = 0.001). Ethanolic extracts (IC_50_ = 166.30 and 27.35 µg DE/mL, respectively; *p* = 0.000) presented the weakest capacity to scavenge ^•^NO. According to the statistical analysis, it seems that phenolic compounds play an important role in ^•^NO scavenging, and phlorotannins play an important role in this behavior (*p* < 0.01).

Therefore, as one of the aims of this research is to compare the antioxidant potential of the *F. vesiculosus* extracts by evaluating their capacity to scavenge different physiological free radicals generated in vitro, acetone 90% extract obtained from the wild *F. vesiculosus* can be identified as the optimal extract, with acetone 90% being the best extraction solvent for both samples.

### 2.3. Enzymes Inhibition

#### 2.3.1. Tyrosinase Inhibition

**Figure 4:**
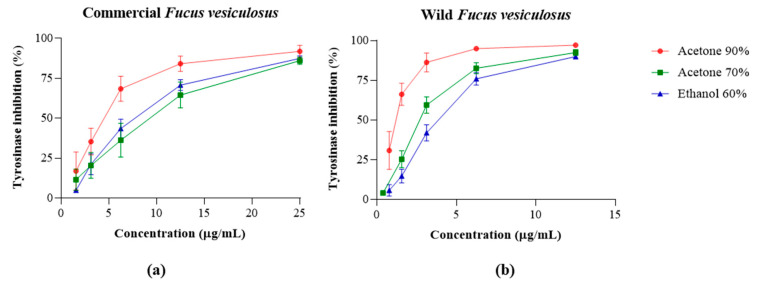
Tyrosinase inhibition of extracts obtained from the commercial (**a**) and wild (**b**) samples of *Fucus vesiculosus*. Values are expressed as the mean ± SD of at least three independent experiments, performed in duplicate.

The tyrosinase inhibition capacities of the commercial and wild *F. vesiculosus* under study are displayed in Figure 4, while the IC_50_ values are presented in Table 4.

Wild *F. vesiculosus* presented higher tyrosinase inhibitory potential compared with the commercial one. Acetone 90% extract obtained from the wild species showed the lowest IC_50_ value (1.23 µg/mL), followed by acetone 70% and ethanol 60% (2.71 and 3.64 µg/mL, respectively). Among the commercial samples, acetone 90% exhibited the highest tyrosinase inhibitory potential (4.19 µg DE/mL) and ethanol 60% the lowest (14.76 µg DE/mL) (*p* < 0.05). Considering wild and commercial samples, there are significant differences between the three extraction solvents mentioned: acetone 90% (*p* = 0.058), acetone 70% (*p* = 0.000), and ethanol 60% (*p* = 0.000). These values were compared with kojic acid, a known skin-lightening ingredient, with an IC_50_ value of 4.09 ± 0.47 µg DE/mL: acetone 90% extract of the wild sample was about three times more effective than the mentioned cosmetic ingredient. In addition, there is a significative negative correlation (*p* < 0.01) between the TPC, phlorotannins, and tyrosinase IC_50_ values, suggesting that these compounds play an important role in tyrosinase inhibition.

#### 2.3.2. Hyaluronidase (HAase) Inhibition

Extracts of the wild *F. vesiculosus* were able to promote the highest HAase inhibition, with acetone 90% presenting the lowest IC_50_ (0.05 mg DE/mL) and ethanol 60% the highest (0.26 mg DE/mL) (*p* < 0.05) (Table 4). Considering wild and commercial samples, there are significant differences in the three extraction solvents mentioned: acetone 90% (*p* = 0.000), acetone 70% (*p* = 0.001), and ethanol 60% (*p* = 0.074).

The IC values obtained with the extracts were compared with that of disodium cromoglicate (DSGC), a natural inhibitor of HAase, with an IC_50_ value of 1.11 ± 0.030 mg DE/mL, all of them presenting greater inhibitory activity than the natural inhibitor. A negative correlation (*p* < 0.01) between TPC, phlorotannins, and HAase inhibition was found, which might point to these compounds as important players in the mentioned biological activity. In fact, the best results obtained with wild *F. vesiculosus* correspond to the extracts with the highest TPC, and phlorotannins consequently.

Therefore, as one of the aims of this research is to determine the ability of *F. vesiculosus* extracts to inhibit key enzymes involved in the skin aging process, acetone 90% extract of wild species can be identified as the optimal extract for both tyrosinase and HAase inhibition, with acetone 90% being the best extraction solvent for both samples.

### 2.4. Anti-Inflammatory Potential

#### 2.4.1. Viability

Cytotoxic effects were screened in RAW 264.7 macrophages for both acetonic and ethanolic extracts of commercial and wild *F. vesiculosus*. For both samples, none of the extracts showed cytotoxic effects under the tested concentrations, with no significant differences compared to the control (*p* > 0.05) for 24 h of exposition, for which all the extracts were considered viable for further analysis (Figure 4).

#### 2.4.2. Effect in LPS-Stimulated RAW 264.7 Cells

**Figure 5:**
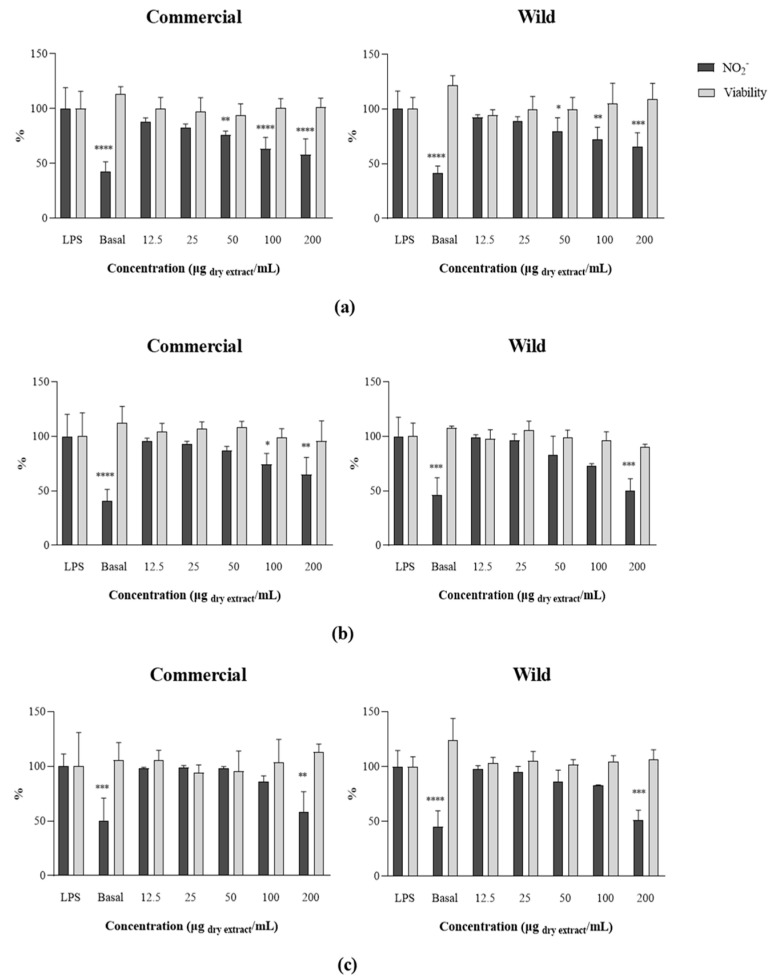
Nitric oxide production by RAW 264.7 cells in the presence of commercial and wild *Fucus vesiculosus* acetone 90% (**a**), acetone 70% (**b**), and ethanol 60% (**c**) extracts, after stimulation with lipopolysaccharide (LPS). Results are expressed as % of nitrite (NO_2_^−^) relative to the control stimulated with LPS. “Basal” represents the nitric oxide produced by RAW 264.7 cells without LPS stimulation. Results are expressed as the mean ± SD of at least three independent assays * *p* < 0.05, ** *p* < 0.01, *** *p* < 0.001, **** *p* < 0.0001 (ANOVA, Tukey HSD, Dunnett HSD).

The anti-inflammatory potential of *F. vesiculosus* extracts was explored in a cell system by determining their capacity to reduce ^•^NO produced by RAW 264.7 cells under LPS stimulation. A pre-exposition to the extracts without LPS stimulation was undertaken to examine the effect of the extracts on basal ^•^NO levels, and then discard any possible pro-inflammatory activity. The extracts alone did not affect the basal ^•^NO levels. Cells were then pre-exposed to serial concentrations of the different extracts obtained from commercial and wild samples and then stimulated with bacterial LPS. After a 22 h stimulation period with LPS, RAW 264.7 exhibited a marked decrease in ^•^NO levels in the culture medium, for the highest concentrations tested, without any toxicity (Figure 5).

As can be seen in Figure 4 and Table 5, the LPS-induced production of ^•^NO decreased in a dose-dependent way in the presence of both commercial and wild samples (12.5 to 200 µg DE/mL), resulting in a decrease in ^•^NO production with the acetone 90% extract to 65.76 and 57.76%, for the wild and commercial *F. vesiculosus* samples, respectively, as compared to the LPS-stimulated control. Both samples had a similar IC_25_ (136.29 ± 65.41 and 88.82 ± 27.75 µg DE/mL, respectively). The acetone 70% extract of the commercial sample was close to achieving IC_50_ for a concentration of 200 µg/mL (50.46% ^•^NO production; IC_25_ = 99.94 ± 22.60 µg DE/mL), closely followed by the acetone 70% extract of the wild sample (64.95% of ^•^NO production; IC_25_ = 146.04 ± 64.17 µg DE/mL). Additionally, cells exposed to ethanolic extracts of wild and commercial samples presented ^•^NO production of 50.97% (IC_25_ = 109.83 ± 12.17 µg DE/mL) and 58.57% (IC_25_ = 148.39 ± 50.73 µg DE/mL) for the highest concentration tested. There were no significant differences between both groups of samples (commercial vs. wild); however, differences can be observed between the different extraction solvents. It is remarkable that a higher phlorotannin content relates to a higher anti-inflammatory potential of the extracts, reaching lower IC_25_ values in acetone 90% extracts, followed by acetone 70% and ethanol 60%. Cell viability did not decrease in any of the concentrations tested, indicating that the extracts do not exert a cytotoxic effect.

## 3. Discussion

### 3.1. Chemical Composition

In order to establish a relationship between the chemical composition and the biological activities, the phytochemical profiles of the samples were established. *F. vesiculosus* extracts were characterized according to their main components (pigments, total phenolic content, and phlorotannins) that resulted from different extractions with solvents of different polarities. The chemical composition showed that wild extracts are richer in both carotenoids and chlorophylls. This can be explained, at least in part, by the sample processing methods; for instance, while the wild samples we explored were lyophilized, commercial samples are normally subjected to less conservative methods and long exposure to oxygen and UV radiation during the drying process, which can contribute to the oxidative degradation of some bioactive metabolites [34]. This can be supported by the absence of chlorophyll-*a* in the commercial sample, which presents higher amounts of chlorophyll derivatives and pheophytin-*a*, a metabolite resulting from the degradation of chlorophyll (Table 1).

Additionally, the time that elapsed between the collection, drying, and analysis of the commercial samples could also affect their chemical profile.

Among all the pigments, fucoxanthin was one of the most representative and the only identified in all the samples. However, there were no significant differences between the different extraction solvents in commercial samples, and their concentration was much lower compared with the extracts obtained from the wild *F. vesiculosus*. Comparing our results obtained with wild *F. vesiculosus* extracts with those of García-Pérez et al. [35] for *Fucus spiralis*, it can be observed that the fucoxanthin content in the ethanolic extracts is closely comparable, with 2046.61 μg/g DE in *F. vesiculosus* and 1171.3 μg/g DE in *F. spiralis*. Regarding chlorophyll-*a*, it was not detected in the ethanolic extracts of either species, whereas in the acetonic extracts, the quantities were comparable, with 225.71 μg/g DE in *F. vesiculosus* and 471.5 μg/g DE in *F. spiralis*. Lastly, β-carotene was not found in any of the extracts from our commercial *F. vesiculosus*, which aligns with the results for *F. spiralis* reported by García-Pérez et al., where this pigment was also absent. Our results are in agreement with Chinnadurai et al. [36], supporting that the acetone extracts of *F. vesiculosus* are dominated by fucoxanthin, which is the pigment responsible for the color of brown algae. Moreover, Silva et al. [37] also highlight the prominence of high concentrations of pheophytin-*a*, which potentially contributes to the olive-brown tonality of *Fucus*, and which was, in parallel with fucoxanthin, one of the dominant pigments identified in our extracts. These studies demonstrate that the extraction method, along with solvent concentration, time of extraction, and temperature, significantly influences both the quantity and type of pigments extracted.

Phlorotannins are the main phenolic compounds in brown algae. Extracts obtained from the wild *F. vesiculosus* presented higher TPC values compared to the commercial ones, with acetone 90% extracts presenting the highest values (1903.83 µg/mg DE). Due to their ability to reduce inflammation and act as antioxidants, phenolic compounds are well-known for their beneficial effects on human health [38,39]. In addition, the efficacy of these compounds is also recognized in the cosmetics field for their ability to overcome hyperpigmentation through the inhibition of the melanin-forming enzyme tyrosinase [40]. Moreover, it is well known that *F. vesiculosus* forms mixed belts in the mid-tide zone and that it has one of the highest phenolic content levels (around 5.80% DW) [41]. Phenolic compounds typically dissolve readily in solvents that are less polar than water, like methanol, ethanol, and acetone, with their aqueous mixtures being most often utilized to extract these chemicals. Many investigations have demonstrated that acetone leads to the highest extraction yields, especially when it comes to phlorotannins [42,43,44,45,46]. Regarding our results in TPhC, the highest values were found for the acetone 90% extract for both commercial and wild *F. vesiculosus*. This is not in accordance with several authors’ findings, who reported that acetone concentrations between 40 and 80% were more successful in producing brown algal extracts with a high phlorotannin content than either pure acetone or water [42,47,48,49]. In the present work, we demonstrate that acetone 90% is the most effective solvent for polyphenols extraction.

### 3.2. Biological Activities

UV radiation is the main cause of skin photoaging by increasing the production of ROS, which leads to the breakdown of extracellular matrix components, oxidative stress, inflammation, and cell apoptosis [50]. Antioxidants, such as pigments or polyphenols present in algae like *Fucus vesiculosus*, act as potent antioxidants and UV filters. This is due to their chemical structure, and in particular, the presence of hydroxyl groups, which enhances both UV absorption and antioxidant activity [51].

The extracts obtained from commercial samples revealed weaker activity than the wild ones, which were significantly more effective in reaching lower IC_50_ values, for the scavenging of the two physiologic free radicals analyzed herein.

Regarding the O_2_^•−^ scavenging assay, our results with the acetone 70% extract of the wild *F. vesiculosus* showed higher O_2_^•−^ scavenging activity (IC_50_ = 0.39 mg DE/mL) than the wild *Fucus spiralis* acetone 70% reported by Ferreres et al. [52] (IC_50_ = 1.30 mg DE/mL). The antioxidant potential observed in our extracts may be attributed to their richness in carotenoids (7082.05 µg/g DE) and polyphenols (1042.75 µg/mg DE), specifically fucoxanthin (7067.12 µg/g DE) and phlorotannins (2.89 µg/mg DE), respectively. In fact, our results agree with those reported by Zaragozá et al. [53], who obtained a negative correlation between TPC and O_2_^•−^ scavenging activity for *F. vesiculosus* extracts. These authors reported a TPC of 163,000 µg/mg DE and O_2_^•−^ scavenging activity of 0.694 mg DE/mL for their ethanolic extract (50–70% ethanol) with fucoxanthin content measured at 1240 µg/g DE. This indicates that while our wild ethanolic extract has lower TPC, it exhibits a higher fucoxanthin concentration and enhanced O_2_^•−^ scavenging ability. Additionally, the negative correlation observed by Lee et al. [54] and Nakai et al. [55] in brown algae further supports the relationship between O_2_^•−^ scavenging and fucoxanthin. This can be corroborated by Miyashita et al. [56], who noted that fucoxanthin has a strong ability to scavenge a variety of free radicals and quench singlet oxygen, suggesting that the higher fucoxanthin content in our extracts may contribute to their improved antioxidant activity. Moreover, fucoxanthin has been shown to have antioxidant, anti-inflammatory, anti-cancer, anti-obesity, anti-diabetic, and hepatoprotective effects [57], which implies that extracts with a high concentration of fucoxanthin have a high potential not only for their application in skincare but also for the treatment of multiple diseases.

In ^•^NO scavenging activity, acetone 90% extracts were the most effective, closely followed by acetone 70% extracts, with ethanolic extracts presenting the weakest activity. These findings suggest that the polarity of the solvent plays an important role in the extraction of bioactive compounds, with the less polar being the most active. This is in agreement with other authors who affirmed that the type of solvent employed in the extraction strongly influences the antioxidant activity, since compounds of different polarities are extracted, leading to different levels of antioxidant potential [58]. Beyond phenolic compounds and based on chemical characterization, fucoxanthin and pheophytin-*a* may significantly contribute to this scavenging activity, taking into account that acetone 90% extracts are considerably richer in pheophytin-*a* and fucoxanthin compared to the other extracts. Consequently, the combined presence of these compounds in the acetone 90% extracts may enhance their ^•^NO scavenging capacity beyond that of phenols alone. Previous studies have demonstrated the radical scavenging potential of fucoxanthin and pheophytin-*a* [59,60,61,62]. It seems that TPC and phlorotannins play an important role in ^•^NO scavenging activity, which is in accordance with different authors [31,63,64,65,66,67] who support the direct relationship between the TPC and antioxidant activity of *F. vesiculosus* extracts. However, these are not the only compounds responsible for ^•^NO scavenging. Previous studies revealed the strong influence of pigments such as chlorophylls and carotenoids as antioxidant components [68,69,70,71], and our extracts are particularly rich in fucoxanthin and pheophytin-*a*, a chlorophyll-*a* derivative. In fact, chlorophylls (*p* < 0.01) and carotenoids (*p* < 0.05) also play an important role in this biological activity. Specifically, fucoxanthin, chlorophyll-*a*, phaeophytin-*a*, and β-carotene presented a negative correlation with ^•^NO scavenging IC_50_. These results are particularly interesting and show the potential of these dry extracts to be used in cosmetics as well as in the treatment of diseases resulting from oxidative stress. This includes diseases associated with inflammation triggered by oxidative stress, which is the cause of many chronic diseases [72].

The primary cause of photoaging is the direct exposure of skin to UV radiation, which lowers the levels of ECM elements such as HA [73]. As these elements are necessary to keep the skin healthy, inhibiting the enzymes that break them down—like collagenase, elastase, and hyaluronidase—constitutes a good approach to slow down the skin aging process. Furthermore, it is crucial to inhibit tyrosinase, an enzyme responsible for melanin production, which is overexpressed with UV radiation, in order to stop excessive pigmentation that is not desired [74]. In the tyrosinase assay, wild *F. vesiculosus* presented higher inhibitory potential compared with the commercial one, with acetone 90% being the best extraction solvent. The different activity in the samples was compared with kojic acid (IC_50_ 4.09 ± 0.47 µg/mL), showing that acetone 90% extract of the wild sample was significantly more effective, presenting an IC_50_ value three times lower than the reference drug (IC_50_ 1.23 ± 0.3 µg DE/mL). In addition, TPC and phlorotannins were highlighted for playing an important role in tyrosinase inhibition. The previous affirmation is supported by several authors who also demonstrated the strong potential linked with the high phenolic compounds (phlorotannins) known for their strong photoprotective, anti-enzymatic, and antioxidant potential [25,26,75,76,77]. In support of this, acetone 70% extracts of *F. vesiculosus* and *F. spiralis* reported by Barbosa et al. [78] have shown tyrosinase IC_50_ values of 2546.82 and 861.73 μg DE/mL, respectively (*p* < 0.0001), with phlorotannin concentrations of 355.80 and 364.07 μg of phloroglucinol equivalents (PhEq)/mg DE, respectively. In comparison, our results indicate significantly stronger inhibition in acetone 70% extracts of commercial and wild *F. vesiculosus*, with IC_50_ values of 8.96 and 2.71 μg DE/mL, respectively. However, the phlorotannin levels in the commercial and wild extracts were notably lower, at 3.02 and 2.89 μg PhEq/mg DE, respectively. Moreover, another study reported that an ethanolic extract of *F. vesiculosus* exhibited a similar tyrosinase inhibition value (IC_50_ = 15.3 µg DE/mL) to our commercial ethanol 60% extract (IC_50_ 14.76 µg DE/mL) [79]. Also, there are different studies of brown algal extracts that are as effective as kojic acid [80,81,82]. In this context, Grina et al. [83] reported an ethanol 70% extract of *F. spiralis* showed a phenolic content of 22.9 µg/mg DE, with a remarkable IC_50_ value for tyrosinase inhibition of 6.19 µg DE/mL. Conversely, in our study, the phenolic content of wild *F. vesiculosus* ethanol 60% extracts showed significantly higher at 792.28 µg/mg DE, yielding an even lower IC_50_ of 3.64 µg DE/mL. These results further underscore the potential of wild *F. vesiculosus*, exhibiting notably superior inhibitory activity compared to both *F. spiralis* and commercial *F. vesiculosus*. Furthermore, based on these two studies, fucoxanthin may play a significant role in tyrosinase inhibition. In vivo studies conducted by Thomas and Kim [84] demonstrated that fucoxanthin isolated from *Laminaria japonica* suppressed tyrosinase activity in ultraviolet B (UV-B)-irradiated guinea pigs and inhibited melanogenesis in UV-B-irradiated mice. Similarly, Shimoda et al. [85] reported that fucoxanthin reduced tyrosinase activity, melanogenesis in melanoma cells, and UVB-induced pigmentation.

As for tyrosinase, wild *F. vesiculosus* exhibited the highest HAase inhibition, with the acetone 90% extract with the lowest IC_50_ value of 0.05 mg DE/mL, followed by the acetone 70% extract with an IC_50_ of 0.16 mg DE/mL. Comparing our findings with existing *F. vesiculosus* studies, the available data are limited. Notably, Pozharitskaya et al. [28] reported that high molecular weight fucoidan isolated from *F. vesiculosus* exhibited a concentration-dependent HAase inhibition with an IC_50_ of 2.9 mg DE/mL. Although this IC_50_ value indicates a lower inhibitory capacity compared to our acetone extracts, it underscores the potential of *F. vesiculosus* components in HAase inhibition. Furthermore, while there are no direct comparisons available with other *F. vesiculosus* studies regarding HAase inhibition, we can refer to the findings of Ferreres et al. [52], who investigated acetone 70% extracts of *Fucus spiralis*. Although *F. vesiculosus* demonstrated lower IC_50_ values, indicating a superior capacity to inhibit Haase, it is crucial to acknowledge the differences between these species. Therefore, our results emphasize the promising potential of *F. vesiculosus* extracts in Haase inhibition while also highlighting the need for further studies focused specifically on this species. There is a relationship between TPC (*r* = −0.837), phlorotannins (*r* = −0.768), and Haase inhibition (*p* < 0.01) which is in agreement with previous reports of brown algae extracts [74,86]. In fact, the better results obtained with wild *F. vesiculosus* are the ones that have the highest TPC, and also phlorotannins as a consequence, considering that these compounds have already been demonstrated to exhibit strong antioxidant and photoprotective potential, as well as the ability to inhibit specific enzymes involved in the skin aging process [25,26,75,76,77].

After stimulation with LPS, RAW 264.7 cells pre-treated with serial dilutions of acetonic and ethanolic *F. vesiculosus* extracts exhibited a marked decrease in ^•^NO without presenting cytotoxic effects. There were no significant differences between both groups of samples (commercial vs. wild); however, differences were observed between the different extraction solvents. It is remarkable that the higher the pigment and phenolic content, the higher the anti-inflammatory potential of the extracts, which led to lower IC_25_ values for the acetone 90% extracts, followed by acetone 70% and ethanol 60%. Additionally, previous studies have shown that the production of inflammatory mediators by macrophages stimulated with LPS can be attenuated by carotenoids [87,88]. Supporting this, Heo et al. [87,89] used fucoxanthin on RAW 264.7 cells stimulated by LPS and found that the expression levels of ^•^NO, TNF-α, and IL-6 were significantly inhibited. This suggests that fucoxanthin may alleviate inflammatory diseases and modulate macrophage activation. The mechanism by which fucoxanthin inhibits ^•^NO production appears to be related to the down-regulation of mRNA and protein expression of the inducible nitric oxide synthase (iNOS), as well as the down-regulation of TNF-α and IL-6 gene expression. Lin et al. [90] demonstrated that pheophytin-*a* can suppress ^•^NO production in LPS-stimulated RAW 264.7 cells, potentially through dual mechanisms involving the inhibition of NOS2 promoter activities and modulation of the ERK and STAT-1 pathways. Although a significant correlation with pheophytin-*a* was not found, a negative correlation (*p* < 0.01) was observed between various chlorophyll derivatives and the anti-inflammatory capacity of the extracts in LPS-stimulated RAW 264.7 cells. This suggests that similar mechanisms may underlie the anti-inflammatory effects observed in this study.

Our results are in accordance with those reported by Barbosa et al. [91], who explored purified phlorotannin extracts from cultivated and wild *F. vesiculosus*, harvested on the north coast of Portugal, which achieved only 25% of inhibition of ^•^NO production by LPS-exposed macrophages at 317 µg DE/mL and 56.5 µg DE/mL, respectively. In the same study, other *Fucus* species were analyzed, showing similar IC_25_ values. *Fucus guirii* G.I. Zardi, K.R. Nicastro, E.S. Serrão and G.A. Pearson, *F.spiralis*, and *F. serratus* showed IC_25_ values of 97.7 µg DE/mL, 95.9 µg/mL, and 77.00 µg DE/mL, respectively, not achieving the 50% of inhibition until the highest concentration tested (500 µg DE/mL). However, Zaragozá et al. [53] reported higher inhibition rates for *F. vesiculosus* (30–35% and 50–70% ethanol) extracts than the ones shown in this study, reaching IC_50_ values in both ethanolic extracts of 95 µg DE/mL and more than 100 µg DE/mL, respectively.

Only a few studies have examined the anti-inflammatory activity of Fucales phlorotannin extracts [92,93,94,95]. Moreover, the currently available work on phlorotannin extracts from *F. vesiculosus* has solely examined how LPS and/or PMA-stimulated macrophages block the release of ^•^NO [53,91,96]. Our results can be explained by the ones reported by Barbosa et al. [91], where samples with equal phlorotannin contents induced a different effect on ^•^NO production and samples with extremely different phlorotannin content demonstrated identical inhibitory capacities. Catarino et al. [97] also showed evidence that the qualitative composition and the complexity of these compounds in the extracts could determine the production of ^•^NO in RAW 264.7 exposed to LPS and, consequently, their anti-inflammatory activity.

Further research should focus on optimizing extraction protocols to enhance the yield and bioactivity of *Fucus vesiculosus* extracts, including the exploration of green solvents and sustainable methods. In vivo studies would be advantageous to validate the antioxidant, anti-inflammatory, and anti-aging effects observed in vitro, with particular attention to safety and efficacy in clinical settings. Additionally, investigating the bioavailability and stability of the bioactive compounds will be crucial for product development. Elucidating the mechanisms of action behind the biological effects of the extracts, through molecular pathway analysis, could deepen our understanding of their therapeutic potential. The impact of environmental factors, geographic location, and harvesting conditions on the chemical composition of *F. vesiculosus* should also be examined to explain the differences between wild and commercial samples. Comparative studies with other marine species may identify alternative sources of bioactive compounds. Finally, developing stable and effective formulations that incorporate *F. vesiculosus* extracts, followed by efficacy testing in real-world applications, will be key to translating these findings into cosmetic and pharmaceutical products.

## 4. Materials and Methods

### 4.1. Sample Collection and Pre-Treatment

The commercial samples of the brown seaweed *F. vesiculosus* were purchased from MORAIS e COSTA & CA. LDA. (Lot: 1011.199.22) and collected on France’s Atlantic coastline, while the wild samples were collected at Praia Norte (41.6970° N, 8.8510° W, Viana do Castelo, Portugal) in February 2024. After collection, fresh seaweeds were cleaned of debris and epiphytes, frozen at −20 °C, and freeze-dried (LyoQuest Plus ECO, Telstar, Barcelona, Spain). Afterward, both algae (commercial and wild) were triturated until we obtained a homogeneous powder. Upon completion of this process, the algae samples were stored in a desiccator until further extraction.

### 4.2. Preparation of Seaweed Extracts

Subsequently, the samples were extracted in duplicate with three different solvents (acetone 90%, acetone 70%, and ethanol 60%). The extractions were performed by adding 6 g of dried seaweed into 30 mL of solvent in each extraction for 1 h at room temperature and with agitation (350 rpm, with a magnetic stirrer Velp AREC). For the preparation of the extracts, an analytical balance (Sartorius Quintix^®^, Göttingen, Germany) with a range between 0.0001 g and 210 g was used for weighting samples. Samples were then centrifuged (3000 rpm, 2 min, 25 °C) (Thermo ScientificTM HERAUS MegafugeTM 16R, Waltham, MA, USA), and the supernatant was filtered with a 0.22 µm pore membrane and evaporated under reduced pressure (BUCHI R-210 Rotary Evaporator, Cambridge, MA, USA). The resulting DE was kept at −20 °C until further chemical and biological analysis.

### 4.3. Phytochemical Characterization

#### 4.3.1. Determination of Pigments Profile by HPLC-PDA

Extracts were resuspended in the same extraction solvent (HPLC-grade) to a final concentration of 25 mg/mL for extracts obtained from wild samples and 50 mg/mL for extracts obtained from commercial samples, and filtered through a 0.22 µm pore membrane before injection. Carotenoid analysis was conducted using a Waters Alliance 2695 high-performance liquid chromatography (HPLC) system with a photodiode array detector (PDA) (USA) for resolution, detection, and identification of the compounds of interest. The stationary phase was a YMC Carotenoid C30 (250 × 4.6 mm; 5 μm) column, kept at a constant temperature (25 °C) with a column heater (Waters Corporation, Milford, CT, USA). The mobile phase consisted of two solvents: methanol (A) and tert-butyl methyl ether (B) (VWR Prolabo), starting with 95% A and initiating a gradient to achieve 10% B at 4 min, 18% B at 19 min, 30% B at 21 min, 50% B from 23 to 29 min, 80% B from 30 to 34 min, and 5% B from 35 to 37 min. The flow rate was 0.90 mL/min, and the injection volume was 10 µL. Data were processed using Empower2 chromatography software (Waters, USA).

Spectral data from all peaks were collected in the range of 200 to 750 nm. Compounds were identified by comparing their retention times and UV-Vis spectra with those of authentic standard solutions. Carotenoid quantification was achieved by measuring the absorbance recorded in the chromatograms relative to external standards at 450 nm. Fucoxanthin, violaxanthin, chlorophyll-*a*, zeaxanthin, and β-carotene were quantified using authentic standards solutions (Extrasynthese, Genay, France; Sigma-Aldrich, St. Louise, MO, USA; DHI, Horsholm, Denmark). Unidentified xanthophylls were quantified using the calibration curve of fucoxanthin, β-carotene derivatives using the calibration curve of β-carotene, and chlorophyll derivatives and pheophytin-*a* using the calibration curve of chlorophyll-*a*. Calibration curves were established with five different concentrations of standards, chosen to represent the range of compound concentrations in the samples. The calibration plots and *r*^2^ values for the analyzed carotenoids and chlorophyll-*a* are presented in Table 6.

#### 4.3.2. Polyphenols Quantification

The total phenolic content (TPC) of *F. vesiculosus* extracts was measured according to the colorimetric assay of Folin–Ciocalteu (Folin and Ciocalteu, 1927), with some modifications previously reported [98]. A standard curve (1) was built using six different concentrations of phloroglucinol (0.015 to 0.5 mg/mL), and the total phenols in each extract were expressed in µg of PhEq per mg of DE, with two independent assays carried out in duplicate.

*y* = 0.002*x* + 0.0086; *r*^2^ = 0.9994 (1)

The phlorotannins content of *F. vesiculosus* extracts was measured according to the 2,4-dimethoxybenzaldehyde (DMBA) assay proposed by (Stern et al., 1996) with some modifications [96]. The absorbance of the sample was determined after 60 min of incubation in the dark at room temperature at 515 nm, using a Synergy HT Multi-detection microplate reader operated by GEN5^TM^ software (Gen5 version, Agilent BioTek, Santa Clara, CA, USA). A standard curve (2) was obtained using seven different concentrations of phloroglucinol (0.003 to 0.25 mg/mL).

*y* = 0.0015*x* + 0.0107; *r*^2^ = 0.992 (2)

Phlorotannins were expressed in µg of PhEq per mg of DE. Two independent assays were carried out in triplicate.

### 4.4. Antioxidant Capacity

#### 4.4.1. ^•^NO Scavenging

Nitric oxide radical (^•^NO) scavenging was measured following the methodology described by Lopes [99] with some modifications. The procedure is based on the determination of the amount of nitrite accumulated in the wells containing extract dilutions after 60 min of incubation. Based on the Griess reaction, equivalent volumes (75 µL) of the incubated mixture and Griess reagent [1:1 mixture (*v*/*v*) of 1% sulfanilamide and 0.1% N-(1-naphthyl) ethylenediamine in 2% H_3_PO_4_] were combined and incubated for 10 min at room temperature in the dark. At 562 nm, the absorbance was measured using a 96-well plate in a Synergy HT Multi-detection microplate reader operated by GEN5^TM^ software. The results were expressed in the percentage of radical scavenging relative control. The results for the calculated IC_50_ values were expressed as the mean ± SD (µg/mL) of at least four independent assays performed in duplicate. The IC_50_ values and corresponding dose–response curves were calculated with Graphpad Prism^®^ software (San Diego, CA, USA; version 8.0.2, for Windows).

#### 4.4.2. Superoxide Anion Radical (O_2_^•−^) Scavenging

Superoxide Anion Radical (O_2_^•−^) scavenging was measured following the methodology described by Lopes [99] with some modifications. All the reagents were dissolved in phosphate buffer (19 µM, pH 7.4). A volume of 50 µL of each extract dilution was mixed with the same volume of NADH solution (166 µM) and 150 µL of NBT (43 µM) in a 96-well plate. After the addition of 50 µL of PMS (2.7 µM), the rate of reaction was monitored with a Synergy HT Multi-detection microplate reader operated by GEN5^TM^ software. The extracts’ scavenging capability was measured by tracking how it influences the reduction of NBT caused by O_2_^•−^ for two minutes, at 562 nm, at RT. The results were expressed in the percentage of radical scavenging relative control. The results for the calculated IC_50_ values were expressed as the mean ± SD (mg/mL) of at least four independent assays performed in duplicate. The IC_50_ values and corresponding dose–response curves were calculated with Graphpad Prism^®^ software.

### 4.5. Enzymatic Assays

#### 4.5.1. Tyrosinase Inhibition

The tyrosinase inhibition assay was performed according to [100] with some modifications. In a 96-well plate, 10 µL of extract serial dilutions were mixed with 90 µL of phosphate buffer (50 mM, pH 6.5) and 50 µL of tyrosinase (50 U/mL in phosphate buffer). Serial dilutions were prepared in DMSO, negative controls were prepared in the absence of extracts, and a positive control was prepared with Kojic acid. The mixture was incubated for 20 min at room temperature. After, 50 µL of L-DOPA (L-3,4-dihydroxyphenylalanine) solution (2.5 mM in phosphate buffer) was added, and the rate of reaction was measured with a Synergy HT Multi-detection microplate reader operated by GEN5^TM^ software, in kinetic mode, at 475 nm. The results for the percentage of tyrosinase inhibition in the presence of *F. vesiculosus* extracts were compared with the untreated control. The results for the calculated IC_50_ values were expressed as the mean ± SD (µg/mL) of at least four independent assays performed in duplicate, and the corresponding dose–response curves were calculated with Graphpad Prism^®^ software.

#### 4.5.2. HAase Inhibition

The hyaluronidase inhibition assay followed the method proposed by [52] with slight modifications. A total of 25 µL of each extract serial dilution was mixed with 175 µL of hyaluronic acid (HA) (0.7 mg/mL) and 25 µL of hyaluronidase (HAase) (900 U/mL in NaCl 0.9%). All the extract serial dilutions were prepared in HCOONa buffer (0.2 M, pH 3.68). After 30 min of incubation at 37 °C, the enzymatic reaction was stopped by adding 25 µL of disodium tetraborate (0.8 M prepared in water: buffer, 5:2 *v*/*v*) solution and heated for 3 min in a water bath at 95 °C. The reaction tubes were cooled down at room temperature before adding 375 µL of DMAB [4-(Dimethylamino) benzaldehyde] 0.67 M (10%) solution. After 20 min of incubation at 37 °C with DMAB, the absorbance of the colored product was measured at 560 nm in a Synergy HT Multi-detection microplate reader operated via GEN5^TM^ software. The negative control was measured in the absence of extract. The results were expressed in the percentage of enzyme inhibition in comparison to the untreated control. The results for the calculated IC_50_ values were expressed as the mean ± SD (mg/mL) of at least three independent assays performed in triplicate, and the corresponding dose–response curves were calculated with Graphpad Prism^®^. Disodium cromoglycate (DSGC) [101] was used as a positive control.

### 4.6. Anti-Inflammatory Potential

#### 4.6.1. Cell Culture and Maintenance

To assess the cytotoxicity and measure the ^•^NO released by RAW 264.7 cells treated with *F. vesiculosus* extracts, an in vitro cytotoxicity assay was performed using this cell line of mouse macrophages (provided by ATCC^®^). Cells were cultured in DMEM Glutamax medium (Dulbecco’s Modified Eagle Medium DMEM GlutaMAXTM-Gibco, Glasgow, UK), supplemented with 10% (*v*/*v*) of fetal bovine serum (Biochrom, Berlin, Germany), 0.1% of amphotericin B (GE Healthcare, Little Chafont, UK), and 1% penicillin–streptomycin (Pen-Strep 100 IU/mL and 10 mg/mL, respectively) (Gibco, Berlin, Germany). Cell maintenance and assays were performed at 37 °C in a 5% CO_2_ humidified atmosphere, and the culture medium was renewed every two days. At 80–90% confluence, cells were detached using a cell scraper to proceed with their seeding or transference. The assays were performed with cell passages between 17 and 20.

#### 4.6.2. Cell Viability—MTT Assay

The MTT assay, previously described in Barbosa et al. [91] with slight modifications, was chosen to assess RAW 246.7 cell viability. Macrophages were seeded in 96-well plates (3.5 × 10^4^ cells/well). Following a 24 h adhesion time, the culture media was removed, and the cells were pre-exposed during 2 h to 100 µL of fresh medium containing the extracts at five different serial doses, ranging from 12.5 to 200 µg/mL. All the extracts were prepared in dimethyl sulfoxide (DMSO, Gibco) and diluted with DMEM prior to cell exposure. The highest concentration of DMSO was not more than 1%, in order to not affect cell viability. After 2 h of pre-incubation with the extracts, cells elicited with LPS were added (final LPS concentration of 1 µg/mL). Then, the cells were maintained under the same conditions for 22 h more. After the incubation period, 75 µL of the cell’s supernatant was transferred to a new 96-well plate in order to determine ^•^NO release and the leftovers of the medium were vacuumed. The cell viability was measured in the original seeded plate by adding 100 µL of MTT solution (0.5 mg/mL in DMEM) following a further incubation of 45 min at 37 °C. The MTT solution was vacuumed, and the formazan salts were dissolved with 100 µL of DMSO. The absorbance of the solubilized formazan salts was quantified spectrophotometrically at 515 nm using a Synergy HT Multi-Detection microplate reader operated via GEN5^TM^ software. Cytotoxicity was expressed as the percentage of cell viability relative to the untreated control.

#### 4.6.3. ^•^NO Release by RAW 264.7 Cells

The accumulated nitrite (NO_2_^−^) in the extracellular media was determined as a measure of ^•^NO generation, as per a previously described technique [91].

As previously mentioned, after 24 h of incubation, 75 µL of culture media were transferred to a new 96-well plate. For measuring the ^•^NO release, 75 µL of Griess reagent was added to the cell’s supernatant and incubated for 10 min at RT in the absence of light. The absorbance of the formed chromophore was read at 562 nm using a Synergy HT Multi-Detection microplate reader operated via GEN5^TM^ software. To deduce the effect of the extracts in the basal ^•^NO generation, and discard a potential pro-inflammatory activity, the production of ^•^NO by RAW 264.7 cells was also evaluated in the presence of the extracts without LPS stimulation.

### 4.7. Statistical Analysis

The statistical analysis was performed using IBM SPSS STATISTICS software, version 27.01.0, IBM Corporation, New York, NY, USA (2020). Data were analyzed for normality and homogeneity of variances using Kolmogorov–Smirnov and Leven’s tests, and then submitted to one-way ANOVA using a Tukey’s HSD (honest significant difference) as a post hoc test, or to a *t*-test. A Pearson correlation test was used to compare normalized expression data between the chemical profile and the biological activities of both wild and commercial *F. vesiculosus*. The results for the calculated IC_50_ and IC_25_ values were expressed as means ± SD (mg/mL or µg/mL) of at least four independent assays, performed in duplicate, for free radicals and enzymatic analysis, and at least three independent assays performed in duplicate, for cell analysis.

## 5. Conclusions

This study highlights the significant influence of the origin of natural samples, as well as the selection of solvents, on the extraction efficiency and bioactivity of *Fucus vesiculosus*. Extracts obtained from wild samples showed superior antioxidant potential compared to commercial ones. Regarding solvent selection, acetone 90% was revealed to be the most effective, resulting in the lowest IC_50_ values for O_2_^•−^ and ^•^NO scavenging activities, emphasizing its efficacy.

Phytochemical analysis revealed significant differences in the content of polyphenols, phlorotannins, and pigments between wild and commercial *F. vesiculosus* samples, with wild extracts showing higher levels. Wild samples, particularly those extracted with 90% acetone, exhibited superior antioxidant and anti-inflammatory activities, as well as enzyme inhibition (tyrosinase and hyaluronidase), relevant to anti-aging properties. These findings suggest that wild *F. vesiculosus* has greater potential for cosmetic applications due to its higher content of bioactive compounds and enhanced biological activities compared to commercial samples. Furthermore, the harvesting of this wild species, which is authorized and controlled by competent authorities and subject to rigorous quality control, makes it a valuable resource in the area of biotechnology.

## Figures and Tables

**Table 1:** Carotenoid and chlorophylls content (µg/g DE) in extracts of commercial and wild *Fucus vesiculosus* obtained with different solvents, determined by HPLC-PDA ^1,2^.

Peak	Compound	RT	Commercial *Fucus vesiculosus* (µg/g DE)			Wild *Fucus vesiculosus* (µg/g DE)		
			Acetone 90%	Acetone 70%	Ethanol 60%	Acetone 90%	Acetone 70%	Ethanol 60%
1	Fucoxanthin	6.20	28.83 ± 1.40 ^d^	16.29 ± 1.38 ^d^	3.24 ± 0.60 ^d^	3857.02 ± 149.06 ^b^	7067.12 ± 302.7 ^a^	2046.61 ± 107.20 ^c^
2	Violaxanthin isomer	9.03	nd	nd	nd	22.07 ± 8.64	nq	nq
3	Chlorophyll-*a*	15.26	nd	nd	nd	225.71 ± 39.86 ^b^	2820.69 ± 106.39 ^a^	nd
4	Zeaxanthin	15.70	nq	nq	nd	nq	nd	nd
5	Chlorophyll derivative	25.30	365.07 ± 1.51 ^a^	60.36 ± 18.71 ^b^	nd	nd	nd	nd
6	Chlorophyll derivative	26.19	73.12 ± 5.00	nq	nd	nd	nd	nd
7	Chlorophyll derivative	27.50	nd	73.84 ± 6.57 ^b^	nd	nd	109.91 ± 6.79 ^a^	nd
8	β-carotene	27.61	107.77 ± 3.03 ^b^	nd	nd	1120.01 ± 156.69 ^a^	14.93 ± 1.49 ^b^	5095.36 ± 14.90 ^a^
9	Pheophytin-*a*	27.85	738.94 ± 24.72 ^c^	89.44 ± 14.19 ^d^	nd	3911.95 ± 221.58 ^b^	482.17 ± 21.05 ^c,d^	nd
10	β-carotene derivative	28.30	22.53 ± 0.31 ^b^	nd	nd	251.07 ± 110.78 ^a^	nd	nd
11	Chlorophyll derivative	28.90	28.03 ± 1.14	nd	nd	nd	nd	nd
12	Chlorophyll derivative	29.21	72.774 ± 13.25	nd	nd	nd	nd	nd
13	Chlorophyll derivative	32.39	312.93 ± 13.79 ^a^	33.95 ± 2.93 ^b^	nd	nd	nd	nd
14	Chlorophyll derivative	33.70	284.22 ± 26.45	nd	nd	nd	nd	nd
15	Chlorophyll derivative	34.50	112.5 ± 12.96	nd	nd	nd	nd	nd
Total Carotenoids			159.13 ± 4.74 ^d^	16.29 ± 1.38 ^d^	3.24 ± 0.6 ^d^	5318.07 ± 329.15 ^b^	7082.05 ± 304.19 ^a^	2046.61 ± 107.2 ^c^
Total Chlorophylls			1987.58 ± 98.82 ^d^	273.70 ± 48.00 ^e^	nd	4137.65 ± 261.44 ^b^	3412.77 ± 134.23 ^c^	5095.36 ± 14.90 ^a^

^1^ Values are expressed as mean ± SD of two determinations. ^2^ Different superscript letters in the same row denote statistical differences at *p* < 0.05 (ANOVA, Tukey HSD, *t*-test). nd: not detected. nq: not quantified. DE, dry extract.

**Table 2:** Total phenolic content (TPC) and Total Phlorotannin Content (TPhC) (µg _PhEq_/mg DE) in *Fucus vesiculosus* extracts obtained with different solvents ^1,2^.

Solvent	TPC (µg _PhEq_/mg DE)		TPhC (µg _PhEq_/mg DE)	
	Commercial	Wild	Commercial	Wild
Acetone 90%	723.48 ± 117.06 ^c,d^	1903.83 ± 53.21 ^a^	5.42 ± 0.20 ^B,C^	15.91 ± 1.22 ^A^
Acetone 70%	500.95 ± 63.85 ^d,e^	1042.75 ± 51.69 ^b^	3.02 ± 0.70 ^C,D^	2.89 ± 0.94 ^D^
Ethanol 60%	361.20 ± 24.32 ^e^	792.28 ± 65.37 ^b,c^	nq	6.77 ± 0.86 ^B^

^1^ Mean ± SD of at least two independent assays. ^2^ Different superscript letters in the same assay denote statistical differences at *p* < 0.05 (ANOVA, Tukey HSD). nq: Not quantified. DE, dry extract. PhEq, phloroglucinol equivalents.

**Table 3:** Half-maximal inhibitory concentration (IC_50_) values obtained for O_2_^•−^ (mg DE/mL) and ^•^NO (µg DE/mL) scavenging, by extracts of commercial and wild *Fucus vesiculosus* obtained with different solvents ^1,2^.

Solvent	O_2_^•−^ Scavenging IC_50_ (mg DE/mL)		^•^NO Scavenging IC_50_ (µg DE/mL)	
	Commercial	Wild	Commercial	Wild
Acetone 90%	1.15 ± 0.06 ^b^	0.30 ± 0.06 ^a^	38.22 ± 9.48 ^B^	12.09 ± 2.71 ^A^
Acetone 70%	1.55 ± 0.19 ^c^	0.39 ± 0.17 ^a^	58.63 ± 6.99 ^C^	29.02 ± 4.72 ^A,B^
Ethanol 60%	1.23 ± 0.18 ^b^	1.59 ± 0.15 ^c^	166.30 ± 15.41 ^D^	27.35 ± 7.98 ^A,B^

^1^ Mean ± SD of at least three independent assays. ^2^ Different superscript letters in the same assay denote statistical differences at *p* < 0.05 (ANOVA, Tukey HSD). DE, dry extract.

**Table 4:** Half-maximal inhibitory concentration (IC_50_) values obtained for tyrosinase (µg DE/mL) and hyaluronidase (HAase) (mg DE/mL) in the presence of different extracts obtained from commercial and wild *Fucus vesiculosus* ^1,2^.

Solvent	Tyrosinase Inhibition IC_50_ (µg DE/mL)		HAase Inhibition IC_50_ (mg DE/mL)	
	Commercial	Wild	Commercial	Wild
Acetone 90%	4.19 ± 0.78 ^b^	1.23 ± 0.3 ^a^	0.26 ± 0.01 ^C,D^	0.05 ± 0.01 ^A^
Acetone 70%	8.96 ± 2.18 ^c^	2.71 ± 0.35 ^a,b^	0.29 ± 0.05 ^D^	0.16 ± 0.01 ^B^
Ethanol 60%	14.76 ± 2.0 ^d^	3.64 ± 0.38 ^a,b^	0.17 ± 0.06 ^B,C^	0.26 ± 0.05 ^C,D^

^1^ Mean ± SD of at least three independent assays. ^2^ Different superscript letters in the same assay denote statistical differences at *p* < 0.05 (ANOVA, Tukey HSD). DE, dry extract.

**Table 5:** Inhibitory concentration (IC_25_) values (µg DE/mL) obtained for nitric oxide (^•^NO) production by LPS-stimulated RAW 264.7 cells in the presence of different extracts of commercial and wild *Fucus vesiculosus* ^1^.

Solvent	^•^NO Production IC_25_ (µg DE/mL)	
	Commercial	Wild
Acetone 90%	88.82 ± 27.75	136.29 ± 65.41
Acetone 70%	99.94 ± 22.60	146.04 ± 64.17
Ethanol 60%	148.39 ± 50.73	109.83 ± 12.17

^1^ Mean ± SD of at least two independent assays. DE, dry extract.

**Table 6:** Calibration curves of authentic standards used for quantification of different carotenoids and chlorophylls.

Standards	Calibration Curve	*r* ^2^
Fucoxanthin	*y* = 108029.9*x* − 4009.9	0.9994
Violaxanthin	*y* = 100034412.5*x* + 83925.9	0.9999
Zeaxanthin	*y* = 273673431.2*x* + 188440.9	0.9895
β-Carotene	*y* = 75843350.4*x* + 2360.1	0.9996
Chlorophyll-*a*	*y* = 12850733.8*x* − 6082.0	0.9943

## Data Availability

Data will be made available upon request to the corresponding authors.

## References

[1] Cheong K.L., Qiu H.M., Du H., Liu Y., Khan B.M. (2018). Oligosaccharides Derived from Red Seaweed: Production, Properties, and Potential Health and Cosmetic Applications. Molecules.

[2] Statista, 2022. Revenue of the Cosmetics Market Worldwide from 2013 to 2026. https://www.statista.com/forecasts/1272313/worldwide-revenue-cosmetics-market-by-segment.

[3] Mordor Intelligence, 2021. Europe Beauty and Personal Care Products Market Report. https://www.mordorintelligence.com/industry-reports/europebeauty-and-personal-care-products-market-industry.

[4] Zion Market Research, 2021. Cosmetic Products Market Size, Share, Growth Report 2030. https://www.zionmarketresearch.com/report/cosmetic-products-market.

[5] Novara A., Sampino S., Paternò F., Keesstra S. (2022). Climate Smart Regenerative Agriculture to Produce Sustainable Beauty Products: The Case Study of Snail Secretion Filtrate (LX360^®^). Sustainability.

[6] Mondello A., Salomone R., Mondello G. (2024). Exploring circular economy in the cosmetic industry: Insights from a literature review. Environ. Impact Assess. Rev..

[7] Ganceviciene R., Liakou A.I., Theodoridis A., Makrantonaki E., Zouboulis C.C. (2012). Skin anti-aging strategies. Derm. -Endocrinol..

[8] Masaki H. (2010). Role of antioxidants in the skin: Anti-aging effects. J. Dermatol. Sci..

[9] Rahal A., Kumar A., Singh V., Yadav B., Tiwari R., Chakraborty S., Dhama K. (2014). Oxidative Stress, Prooxidants, and Antioxidants: The Interplay. BioMed Res. Int..

[10] Dunaway S., Odin R., Zhou L., Ji L., Zhang Y., Kadekaro A.L. (2018). Natural Antioxidants: Multiple Mechanisms to Protect Skin from Solar Radiation. Front.Pharmacol..

[11] Wojcik M., Burzynska-Pedziwiatr I., Wozniak L.A. (2010). A Review of Natural and Synthetic Antioxidants Important for Health and Longevity. Curr. Med. Chem..

[12] López-Hortas L., Flórez-Fernández N., Torres M.D., Ferreira-Anta T., Casas M.P., Balboa E.M., Falqué E., Domínguez H. (2021). Applying Seaweed Compounds in Cosmetics, Cosmeceuticals and Nutricosmetics. Mar. Drugs.

[13] Khan A.D., Alam M.N. (2019). Cosmetics and their Associated Adverse Effects: A Review. JAPSR.

[14] Morais T., Cotas J., Pacheco D., Pereira L. (2021). Seaweeds Compounds: An Ecosustainable Source of Cosmetic Ingredients?. Cosmetics.

[15] Thiyagarasaiyar K., Goh B.H., Jeon Y.J., Yow Y.Y. (2020). Algae Metabolites in Cosmeceutical: An Overview of Current Applications and Challenges. Mar. Drugs.

[16] Jesumani V., Du H., Aslam M., Pei P., Huang N. (2019). Potential Use of Seaweed Bioactive Compounds in Skincare—A Review. Mar. Drugs.

[17] Alhajj M.J., Montero N., Yarce C.J., Salamanca C.H. (2020). Lecithins from Vegetable, Land, and Marine Animal Sources and Their Potential Applications for Cosmetic, Food, and Pharmaceutical Sectors. Cosmetics.

[18] Couteau C., Coiffard L. (2020). Phycocosmetics and Other Marine Cosmetics, Specific Cosmetics Formulated Using Marine Resources. Mar. Drugs.

[19] Pereira A.G., Otero P., Echave J., Carreira-Casais A., Chamorro F., Collazo N., Jaboui A., Lourenço-Lopes C., Simal-Gandara J., Prieto M.A. (2021). Xanthophylls from the Sea: Algae as Source of Bioactive Carotenoids. Mar. Drugs.

[20] Ferreira M.S., Resende D.I.S.P., Lobo J.M.S., Sousa E., Almeida I.F. (2021). Marine Ingredients for Sensitive Skin: Market Overview. Mar. Drugs.

[21] Kim S.K., Ravichandran Y.D., Khan S.B., Kim Y.T. (2008). Prospective of the cosmeceuticals derived from marine organisms. Biotechnol. Bioprocess Eng..

[22] Naser W. (2021). The cosmetic effects of various natural biofunctional ingredients against skin aging: A review. Int. J. App. Pharm..

[23] Gil T.Y., Kang Y.M., Eom Y.J., Hong C.H., An H.J. (2019). Anti-Atopic Dermatitis Effect of Seaweed *Fulvescens* Extract via Inhibiting the STAT1 Pathway. Mediat. Inflamm..

[24] Takahashi M., Takahashi K., Abe S., Yamada K., Suzuki M., Masahisa M., Endo M., Abe K., Inoue R., Hoshi H. (2020). Improvement of Psoriasis by Alteration of the Gut Environment by Oral Administration of Fucoidan from *Cladosiphon okamuranus*. Mar. Drugs.

[25] Pereira L. (2018). Seaweeds as Source of Bioactive Substances and Skin Care Therapy—Cosmeceuticals, Algotheraphy, and Thalassotherapy. Cosmetics.

[26] Freitas R., Martins A., Silva J., Alves C., Pinteus S., Alves J., Teodoro F., Ribeiro H.M., Gonçalves L., Petrovski Ž. (2020). Highlighting the Biological Potential of the Brown Seaweed *Fucus spiralis* for Skin Applications. Antioxidants.

[27] Rodríguez-Bernaldo de Quirós A., Frecha-Ferreiro S., Vidal-Pérez A.M., López-Hernández J. (2010). Antioxidant compounds in edible brown seaweeds. Eur. Food Res. Technol..

[28] Pozharitskaya O.N., Obluchinskaya E.D., Shikov A.N. (2020). Mechanisms of Bioactivities of Fucoidan from the Brown Seaweed *Fucus vesiculosus* L. of the Barents Sea. Mar. Drugs.

[29] Hermund D.B., Torsteinsen H., Vega J., Figueroa F.L., Jacobsen C. (2022). Screening for New Cosmeceuticals from Brown Algae *Fucus vesiculosus* with Antioxidant and Photo-Protecting Properties. Mar. Drugs.

[30] Golshany H., Siddiquy M., Elbarbary A., Seddiek A.S., Kamal A., Yu Q., Fan L. (2024). Exploring *Fucus vesiculosus* phlorotannins: Insights into chemistry, extraction, purification, identification and bioactivity. Food Biosci..

[31] Fitton J.H., Dell’Acqua G., Gardiner V.-A., Karpiniec S.S., Stringer D.N., Davis E. (2015). Topical Benefits of Two Fucoidan-Rich Extracts from Marine Macroalgae. Cosmetics.

[32] Marinho G.S., Sørensen A.D.M., Safafar H., Pedersen A.H., Holdt S.L. (2018). Antioxidant content and activity of the seaweed *Saccharina latissima*: A seasonal perspective. J. App Phycol..

[33] Konstantin B., Anastasia P., Nikolay I., Daria P. (2023). Seasonal variations in the chemical composition of Arctic brown macroalgae. Algal. Res..

[34] Groeneveld I., Kanelli M., Ariese F., Van Bommel M.R. (2023). Parameters that affect the photodegradation of dyes and pigments in solution and on substrate–An overview. Dyes Pigment..

[35] Garcia-Perez P., Lourenço-Lopes C., Silva A., Pereira A.G., Fraga-Corral M., Zhao C., Xiao J., Simal-Gandara J., Prieto M.A. (2022). Pigment composition of nine brown algae from the Iberian northwestern coastline: Influence of the extraction solvent. Mar. Drugs.

[36] Chinnadurai S., Kalyanasundaram G., Chermapandi P., Annadurai H.A., Perumal A. (2013). Estimation of major pigment content in seaweeds collected from Pondicherry coast. Int. J. Sci. Technol..

[37] Silva A.F.R., Abreu H., Silva A.M.S., Cardoso S.M. (2019). Effect of Oven-Drying on the Recovery of Valuable Compounds from *Ulva rigida*, *Gracilaria* sp. and *Fucus vesiculosus*. Mar. Drugs.

[38] Boo Y.C. (2019). Can Plant Phenolic Compounds Protect the Skin from Airborne Particulate Matter?. Antioxidants.

[39] Rahman M.M., Rahaman M.S., Islam M.R., Rahman F., Mithi F.M., Alqahtani T., Almikhlafi M.A., Alghamdi S.Q., Alruwaili A.S., Hossain M.S. (2022). Role of Phenolic Compounds in Human Disease: Current Knowledge and Future Prospects. Molecules.

[40] Erdogan Orhan I., Tareq Hassan Khan M. (2014). Flavonoid Derivatives as Potent Tyrosinase Inhibitors—A Survey of Recent Findings Between 2008–2013. Curr. Top. Med. Chem..

[41] Connan S., Goulard F., Stiger V., Deslandes E., Gall E.A. (2004). Interspecific and temporal variation in phlorotannin levels in an assemblage of brown algae. Bot. Mar..

[42] Koivikko R., Loponen J., Honkanen T., Jormalainen V. (2005). Contents of soluble, cell-wall-bound and exuded phlorotannins in the brown alga *Fucus vesiculosus*, with implications on their ecological functions. J. Chem. Ecol..

[43] Kim A.R., Shin T.S., Lee M.S., Park J.Y., Park K.E., Yoon N.Y., Kim J.S., Choi J.S., Jang B.C., Byun D.S. (2009). Isolation and Identification of Phlorotannins from *Ecklonia stolonifera* with Antioxidant and Anti-inflammatory Properties. J. Agric. Food Chem..

[44] Breton F., Cérantola S., Gall E.A. (2011). Distribution and radical scavenging activity of phenols in *Ascophyllum nodosum* (Phaeophyceae). J. Exp. Mar. Biol. Ecol..

[45] Liu H., Gu L. (2012). Phlorotannins from Brown Algae (*Fucus vesiculosus*) Inhibited the Formation of Advanced Glycation Endproducts by Scavenging Reactive Carbonyls. J. Agric. Food Chem..

[46] Honold P.J., Jacobsen C., Jónsdóttir R., Kristinsson H.G., Hermund D.B. (2016). Potential seaweed-based food ingredients to inhibit lipid oxidation in fish-oil-enriched mayonnaise. Eur. Food Res. Tech..

[47] Auezova L., Najjar F., Selivanova O., Hajj Moussa E., Diab Assaf M. (2013). Antioxidant activity of brown alga *Saccharina bongardiana* from Kamchatka (Pacific coast of Russia). A methodological approach. J. Appl. Phycol..

[48] Tierney M.S., Smyth T.J., Hayes M., Soler-Vila A., Croft A.K., Brunton N. (2013). Influence of pressurised liquid extraction and solid–liquid extraction methods on the phenolic content and antioxidant activities of Irish macroalgae. Int. J. Food Sci. Technol..

[49] Belda M., Sanchez D., Bover E., Prieto B., Padrón C., Cejalvo D., Lloris J.M. (2016). Extraction of polyphenols in *Himanthalia elongata* and determination by high performance liquid chromatography with diode array detector prior to its potential use against oxidative stress. J. Chromatogr. B..

[50] Sharma P., Dhiman T., Negi R.S., Anshad O.C., Gupta K., Bhatti J.S., Thareja S. (2024). A comprehensive review of the molecular mechanisms driving skin photoaging and the recent advances in therapeutic interventions involving natural polyphenols. S. Afr. J. Bot..

[51] Nunes A.R., Vieira G.P., Queiroz D.B., Leal A.L.A.B., Morais S.M., Muniz D.F., Calixto-Junior J.T., Coutinho H.D.M. (2018). Use of flavonoids and cinnamates, the main photoprotectors with natural origin. Adv. Pharmacol. Pharm. Sci..

[52] Ferreres F., Lopes G., Gil-Izquierdo A., Andrade P., Sousa C., Mouga T., Valentão P. (2012). Phlorotannin Extracts from Fucales Characterized by HPLC-DAD-ESI-MSn: Approaches to Hyaluronidase Inhibitory Capacity and Antioxidant Properties. Mar. Drugs.

[53] Zaragozá M.C., López D., Sáiz M.P., Poquet M., Pérez J., Puig-Parellada P., Màrmol F., Simonetti P., Gardana C., Lerat Y. (2008). Toxicity and Antioxidant Activity in Vitro and in Vivo of Two Fucus vesiculosus Extracts. J. Agric. Food Chem..

[54] Lee S.M., Na M.K., An R.B., Min B.S., Lee H.K. (2003). Antioxidant Activity of Two Phloroglucinol Derivatives from Dryopteris crassirhizoma. Biol. Pharm. Bull..

[55] Nakai M., Kageyama N., Nakahara K., Miki W. (2006). Phlorotannins as Radical Scavengers from the Extract of *Sargassum ringgoldianum*. Mar. Biotechnol..

[56] Miyashita K., Beppu F., Hosokawa M., Liu X., Wang S. (2020). Nutraceutical characteristics of the brown seaweed carotenoid fucoxanthin. Arch. Biochem. Biophys..

[57] Bae M., Kim M.B., Park Y.K., Lee J.Y. (2020). Health benefits of fucoxanthin in the prevention of chronic diseases. Biochimica et Biophysica Acta (BBA). Mol. Cell Biol. Lipids..

[58] Radhika D., Veerabahu C., Priya R. (2013). In vitro studies on antioxidant and haemagglutination activity of some selected seaweeds. Int. J. Pharm Pharm. Sci..

[59] Ayyad S.E.N., Ezmirly S.T., Basaif S.A., Alarif W.M., Badria A.F., Badria F.A. (2011). Antioxidant, cytotoxic, antitumor, and protective DNA damage metabolites from the red sea brown alga *Sargassum* sp. Pharmacogn. Res..

[60] Ragubeer N., Limson J.L., Beukes D.R. (2012). Electrochemistry-guided isolation of antioxidant metabolites from *Sargassum elegans*. Food Chem..

[61] Balboa E.M., Conde E., Moure A., Falqué E., Domínguez H. (2013). In vitro antioxidant properties of crude extracts and compounds from brown algae. Food Chem..

[62] Hsu C.Y., Chao P.Y., Hu S.P., Yang C.M. (2013). The Antioxidant and Free Radical Scavenging Activities of Chlorophylls and Pheophytins. Food Nutr. Sci..

[63] Jiménez-Escrig A., Jiménez-Jiménez I., Pulido R., Saura-Calixto F. (2001). Antioxidant activity of fresh and processed edible seaweeds. J. Sci. Food Agric..

[64] Díaz-Rubio M.E., Pérez-Jiménez J., Saura-Calixto F. (2009). Dietary fiber and antioxidant capacity in *Fucus vesiculosus* products. Int. J. Food Sci. Nutr.

[65] Wang T., Jónsdóttir R., Liu H., Gu L., Kristinsson H.G., Raghavan S., Ólafsdóttir G. (2012). Antioxidant Capacities of Phlorotannins Extracted from the Brown Algae *Fucus vesiculosus*. J. Agric. Food Chem..

[66] Liu X., Yuan W., Sharma-Shivappa R., van Zanten J. (2017). Antioxidant activity of phlorotannins from brown algae. Int. J. Agric. Biol. Eng..

[67] Agregán R., Munekata P.E.S., Franco D., Carballo J., Barba F.J., Lorenzo J.M. (2018). Antioxidant Potential of Extracts Obtained from Macro- (*Ascophyllum nodosum*, *Fucus vesiculosus* and *Bifurcaria bifurcata*) and Micro-Algae (*Chlorella vulgaris* and *Spirulina platensis*) Assisted by Ultrasound. Medicines.

[68] Maoka T., Fujiwara Y., Hashimoto K., Akimoto N. (2002). Rapid Identification of Carotenoids in a Combination of Liquid Chromatography / UV-Visible Absorption Spectrometry by Photodiode-Array Detector and Atomospheric Pressure Chemical Ionization Mass Spectrometry (LC/PAD/APCI-MS). J. Oleo Sci..

[69] Lanfer-Marquez U.M., Barros R.M.C., Sinnecker P. (2005). Antioxidant activity of chlorophylls and their derivatives. Food Res. Int..

[70] Rajauria G., Foley B., Abu-Ghannam N. (2017). Characterization of dietary fucoxanthin from *Himanthalia elongata* brown seaweed. Food Res. Int..

[71] Rajauria G. (2019). In-Vitro Antioxidant Properties of Lipophilic Antioxidant Compounds from 3 Brown Seaweed. Antioxidants.

[72] Hussain T., Tan B., Yin Y., Blachier F., Tossou M.C., Rahu N. (2016). Oxidative stress and inflammation: What polyphenols can do for us?. Oxid. Med. Cell. Longev..

[73] Paliwal S., Fagien S., Sun X., Holt T., Kim T., Hee C.K., Van Epps D., Messina D.J. (2014). Skin Extracellular Matrix Stimulation following Injection of a Hyaluronic Acid–Based Dermal Filler in a Rat Model. Plast. Reconstr. Surg..

[74] Susano P., Silva J., Alves C., Martins A., Gaspar H., Pinteus S., Mouga T., Goettert M.I., Petrovski Ž., Branco L.B. (2021). Unravelling the Dermatological Potential of the Brown Seaweed *Carpomitra costata*. Mar. Drugs.

[75] Moreira L.C., de Ávila R.I., Veloso D.F.M.C., Pedrosa T.N., Lima E.S., do Couto R.O., Lima E.M., Batista A.C., de Paula J.R., Valadares M.C. (2017). In vitro safety and efficacy evaluations of a complex botanical mixture of *Eugenia dysenterica* DC. (Myrtaceae): Prospects for developing a new dermocosmetic product. Toxicol. Vitr..

[76] Załuski D., Olech M., Kuźniewski R., Verpoorte R., Nowak R., Smolarz H.D. (2017). LC-ESI-MS/MS profiling of phenolics from *Eleutherococcus* spp. inflorescences, structure-activity relationship as antioxidants, inhibitors of hyaluronidase and acetylcholinesterase. Saudi Pharm. J..

[77] Rosa G.P., Barreto M.C., Seca A.M.L. (2019). Pharmacological effects of *Fucus spiralis* extracts and phycochemicals: A comprehensive review. Botanica Marina.

[78] Barbosa M., Valentão P., Ferreres F., Gil-Izquierdo Á., Andrade P.B. (2020). In vitro multifunctionality of phlorotannin extracts from edible *Fucus* species on targets underpinning neurodegeneration. Food Chem..

[79] Soares C., Paíga P., Marques M., Neto T., Carvalho A.P., Paiva A., Simões P., Costa L., Bernardo A., Fernández N. (2021). Multi-Step Subcritical Water Extracts of *Fucus vesiculosus* L. and *Codium tomentosum* Stackhouse: Composition, Health-Benefits and Safety. Processes.

[80] Arguelles E.D.L.R., Sapin A.B. (2020). Bioactive properties of *Sargassum siliquosum* J. Agardh (Fucales, Ochrophyta) and its potential as source of skin-lightening active ingredient for cosmetic application. J. Appl. Pharma. Sci..

[81] Arguelles E.D.L.R., Sapin A.B. (2020). Bioprospecting of *Turbinaria ornata* (Fucales, phaeophyceae) for cosmetic application: Antioxidant, tyrosinase inhibition and antibacterial activities. J. Int. Soc. Southeast Asian Agric. Sci..

[82] Arguelles E.D.L.R. (2021). Evaluation of Antioxidant Capacity, Tyrosinase Inhibition, and Antibacterial Activities of Brown Seaweed, *Sargassum ilicifolium* (Turner) C. Agardh 1820 for Cosmeceutical Application. J. Fish. Environ..

[83] Grina F., Ullah Z., Kaplaner E., Moujahid A., Eddoha R., Nasser B., Essamadi A. (2020). In vitro enzyme inhibitory properties, antioxidant activities, and phytochemical fingerprints of five Moroccan seaweeds. S. Afri. J. Bot..

[84] Thomas N., Kim S. (2013). Beneficial effects of marine algal compounds in cosmeceuticals. Mar. Drugs.

[85] Shimoda H., Tanaka J., Shan S.J., Maoka T. (2010). Anti-pigmentary activity of fucoxanthin and its influence on skin mRNA expression of melanogenic molecules. J. Pharm. Pharmacol..

[86] Arunkumar K., Raj R., Raja R., Carvalho I.S. (2021). Brown seaweeds as a source of anti-hyaluronidase compounds. S. Afri. J. Bot..

[87] Heo S.J., Yoon W.J., Kim K.N., Ahn G.N., Kang S.M., Kang D.H., Affan A., Oh C., Jung W.K., Jeon Y.J. (2010). Evaluation of anti-inflammatory effect of fucoxanthin isolated from brown algae in lipopolysaccharide-stimulated RAW 264.7 macrophages. Food Chem. Toxic..

[88] Kim K.N., Heo S.J., Yoon W.J., Kang S.M., Ahn G., Yi T.H., Jeon Y.J. (2010). Fucoxanthin inhibits the inflammatory response by suppressing the activation of NF-κB and MAPKs in lipopolysaccharide-induced RAW 264.7 macrophages. Eur. J. Pharmacol..

[89] Heo S.J., Yoon W.J., Kim K.N., Oh C., Choi Y.U., Yoon K.T., Kang D.H., Qian Z.J., Choi I.W., Jung W.K. (2012). Anti-inflammatory effect of fucoxanthin derivatives isolated from *Sargassum siliquastrum* in lipopolysaccharide-stimulated RAW 264.7 macrophage. Food Chem. Toxicol..

[90] Lin C.Y., Lee C.H., Chang Y.W., Wang H.M., Chen C.Y., Chen Y.H. (2014). Pheophytin a inhibits inflammation via suppression of LPS-induced nitric oxide synthase-2, prostaglandin E2, and interleukin-1β of macrophages. Int. J. Mol. Sci..

[91] Barbosa M., Lopes G., Ferreres F., Andrade P.B., Pereira D.M., Gil-Izquierdo Á., Valentão P. (2017). Phlorotannin extracts from Fucales: Marine polyphenols as bioregulators engaged in inflammation-related mediators and enzymes. Algal Res..

[92] Dutot M., Fagon R., Hemon M., Rat P. (2012). Antioxidant, Anti-inflammatory, and Anti-senescence Activities of a Phlorotannin-Rich Natural Extract from Brown Seaweed *Ascophyllum nodosum*. App. Biochem. Biotechnol..

[93] Kellogg J., Esposito D., Grace M.H., Komarnytsky S., Lila M.A. (2015). Alaskan seaweeds lower inflammation in RAW 264.7 macrophages and decrease lipid accumulation in 3T3-L1 adipocytes. J. Funct. Foods.

[94] Tamanai-Shacoori Z., Chandad F., Rébillard A., Cillard J., Bonnaure-Mallet M. (2014). Silver-Zeolite Combined to Polyphenol-Rich Extracts of *Ascophyllum nodosum*: Potential Active Role in Prevention of Periodontal Diseases. PLoS ONE.

[95] Bahar B., O’Doherty J.V., Smyth T.J., Sweeney T. (2016). A comparison of the effects of an *Ascophyllum nodosum* ethanol extract and its molecular weight fractions on the inflammatory immune gene expression in-vitro and ex-vivo. Innov. Food Sci. Emerg. Technol..

[96] Lopes G., Sousa C., Silva L.R., Pinto E., Andrade P.B., Bernardo J., Mouga T., Valentão P. (2012). Can Phlorotannins Purified Extracts Constitute a Novel Pharmacological Alternative for Microbial Infections with Associated Inflammatory Conditions?. PLoS ONE.

[97] Catarino M.D., Silva A., Cruz M.T., Mateus N., Silva A.M.S., Cardoso S.M. (2020). Phlorotannins from *Fucus vesiculosus*: Modulation of Inflammatory Response by Blocking NF-κB Signaling Pathway. Int. J. Mol. Sci..

[98] Morone J., Lopes G., Preto M., Vasconcelos V., Martins R. (2020). Exploitation of Filamentous and Picoplanktonic Cyanobacteria for Cosmetic Applications: Potential to Improve Skin Structure and Preserve Dermal Matrix Components. Mar. Drugs.

[99] Lopes G. (2014). Seaweeds from the Portuguese Coast: Chemistry, Antimicrobial and Anti-Inflammatory Capacity. Ph.D. Dissertation.

[100] Adhikari A., Devkota H.P., Takano A., Masuda K., Nakane T., Basnet P., Skalko-Basnet N. (2008). Screening of Nepalese crude drugs traditionally used to treat hyperpigmentation: In vitro tyrosinase inhibition. Int. J. Cos. Sci..

[101] Barbosa M., Lopes G., Valentão P., Ferreres F., Gil-Izquierdo Á., Pereira D.M., Andrade P.B. (2018). Edible seaweeds’ phlorotannins in allergy: A natural multi-target approach. Food Chem..

